# Using SRAM Based FPGAs for Power-Aware High Performance Wireless Sensor Networks

**DOI:** 10.3390/s120302667

**Published:** 2012-02-28

**Authors:** Juan Valverde, Andres Otero, Miguel Lopez, Jorge Portilla, Eduardo de la Torre, Teresa Riesgo

**Affiliations:** Centro de Electronica Industrial, Universidad Politecnica de Madrid, Jose Gutierrez Abascal 2, Madrid 28006, Spain; E-Mails: joseandres.otero@upm.es (A.O.); miguel.lopezl@alumnos.upm.es (M.L.); jorge.portilla@upm.es (J.P.); eduardo.delatorre@upm.es (E.T.); teresa.riesgo@upm.es (T.R.)

**Keywords:** wireless sensor networks (WSNs), FPGA, dynamic and partial reconfiguration (DPR), energy efficiency

## Abstract

While for years traditional wireless sensor nodes have been based on ultra-low power microcontrollers with sufficient but limited computing power, the complexity and number of tasks of today’s applications are constantly increasing. Increasing the node duty cycle is not feasible in all cases, so in many cases more computing power is required. This extra computing power may be achieved by either more powerful microcontrollers, though more power consumption or, in general, any solution capable of accelerating task execution. At this point, the use of hardware based, and in particular FPGA solutions, might appear as a candidate technology, since though power use is higher compared with lower power devices, execution time is reduced, so energy could be reduced overall. In order to demonstrate this, an innovative WSN node architecture is proposed. This architecture is based on a high performance high capacity state-of-the-art FPGA, which combines the advantages of the intrinsic acceleration provided by the parallelism of hardware devices, the use of partial reconfiguration capabilities, as well as a careful power-aware management system, to show that energy savings for certain higher-end applications can be achieved. Finally, comprehensive tests have been done to validate the platform in terms of performance and power consumption, to proof that better energy efficiency compared to processor based solutions can be achieved, for instance, when encryption is imposed by the application requirements.

## 1. Introduction

WSNs have evolved dramatically since the first platform turned up ten years ago. Traditional applications are still being studied and improved while, at the same time, new approaches have appeared. So far, the most common applications were related to environmental care, agriculture [1] or hazardous environment monitoring. Normally, these applications share some features such as low data rates, non-restrictive latency and low number of nodes, among others. For these reasons, the processing requirements per node are low, so tiny processors can be used. These low profile processors offer enough processing capabilities while offering ultra-low power consumption. The MSP430 microcontroller from Texas Instruments included in the TelosB [2] platform from the University of California at Berkeley or the ATmega1281 used by Libelium in the Waspmote is an example of these.

On the contrary, as technology has evolved, different scenarios that require much more powerful processing units have appeared. As an example, traditional applications that require more nodes to cover wider areas have to deal with bigger amounts of raw data while latency remains a crucial matter. In addition, new applications such as video and image processing, have appeared within the WSN field, where the need of carrying out data compression or encryption algorithms also requires the use of much more powerful processors with higher power consumption. Due to these energy demands, new design requirements must be taken into account. Higher processing speed, larger memory, etc., are needed, where power consumption is still one of the main concerns for the WSN designer. This work provides the complete design of a new much more powerful processing layer for the Cookies, a wireless sensor node proposed by the authors (detailed in [3]), in order to adapt the platform to the new high performance application requirements.

One solution could be the inclusion of more powerful microcontrollers [4]. This might be suitable for some applications, since they offer a good trade-off between price, size and programming flexibility. Nevertheless, when facing very intensive tasks, the computing time can be very high leading to a non-efficient solution in terms of energy consumption. A better solution can be achieved using Digital Signal Processors (DSPs). However, even though the computing time can be reduced compared to a standard microcontroller, it is still very high, so it is difficult to keep power consumption at acceptable levels for WSNs.

On the other hand, novel solutions can be achieved considering the specific features of WSN applications. Thus, the standard working profile of a WSN has a very low duty cycle (1%–10%). The node is awake only during the time concerning for carrying out measurements and doing some operations [5–7]. Then, the node is taken to a power down or sleep mode where power consumption should be close to zero. Due to this reason, very quick processing engines are very suitable for these working profiles leading to very efficient solutions in terms of energy consumption by reducing processing time.

**Figure 1.**
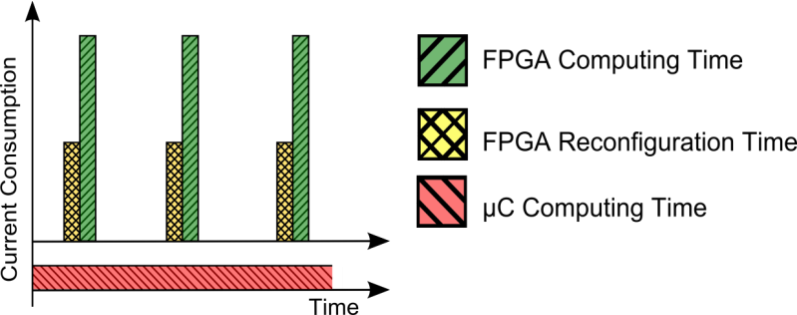
Consumption profile comparison.

The best way to provide this very high speed processing capabilities is the use of hardware based systems. FPGAs and ASICs share the capability of doing tasks in parallel. This fact can decrease the computing time sharply so the node can be in sleep mode for longer periods of time. However, the use of ASICs in WSNs is not always suitable due to the lack of flexibility and the huge design time and cost, at least in the prototyping stage. Due to the reasons above, FPGAs are presented as a very suitable solution for high performance WSN applications. In Figure 1, a theoretical comparison of the working profile between an FPGA and a microcontroller is shown.

While the time needed by the FPGA to carry out a certain task usually falls a long way short of the time needed by purely software based solutions, the main drawback is the fact that FPGA configuration time (time used to configure the device after a sleeping period) is significant compared to the computing time. One of the main contributions of this work is to prove that for an actual high-performance WSN node implementation the addition of both configuration and processing times is still lower than the time the microcontroller requires to carry out the same task. This assertion is only true when dealing with complex algorithms where the computing time needed for the microcontroller is bigger than the configuration time the FPGA needs. Moreover, the configuration time of the FPGA depends on the size of the bitstream file (file with information about the configuration) and it can vary from one application to another. In this paper the possibility of compressing bitstream files or loading partial ones is also exploited.

Furthermore, the use of FPGAs allows Partial and Dynamic Reconfiguration (DPR) which has many advantages when working with wireless sensor nodes. The possibility of adapting the system functionality loading new blocks at run time in a very fast way, when working with changeable environments, opens up new opportunities. Moreover, the reduction in size of a partial bitstream allows the file to be easily sent through the network via radio. The capability of configuring only the needed hardware blocks allows the implementation of energy saving methodologies such as start-up sequences [8], fast configuration, *etc*.

The implementation of these techniques may not be enough to reach acceptable values of power consumption. Even though the FPGA may be working in a very efficient way, the rest of the components will consume energy even when they are not needed. In this work, a design divided into seven different power islands that can be powered separately is presented. In this way, only the components needed are powered while the rest remain powered off.

It is not the aim of this work to compare our proposed solution with ultra-low power WSN platforms since the application scope is completely different. This way, a trade-off between low energy consumption and high performance capabilities must be found. Rather, the main goal of this work is to show that by using both hardware acceleration and power management strategies, it is possible to obtain high performance nodes with power consumption levels in the range of WSN applications.

The rest of the paper is organized as follows: in Section 2, the state-of-the-art is reviewed, while Section 3 deals with all the details of the proposed architecture. In Section 4 different power management strategies are discussed. In Section 5 all the results and measurements are shown to illustrate the platform validation. To finish, some conclusions are given and future work proposed.

## 2. Related Work

The complexity of the applications faced by WSNs is increasing, changing node requirements accordingly. This is the context where high performance emerges as an enabling technology for WSNs. Most of these scenarios are driven by the integration of multimedia sensors like low-power video cameras or microphones. WSNs that include video, audio and still image processing capabilities are known as Wireless Multimedia Sensor Networks (WMSN). Surveys concerning WMSN architectures are offered in [9,10]. According to [10], WMSN applications can be classified into five groups: Surveillance, Traffic Monitoring and Enforcement, Personal and Health Care, Gaming and Environmental and finally, Industrial. Surveillance is an example of a traditional application field where the increase of node processing capabilities can lead to revolutionary changes on the applications. While traditional approaches were limited to the detection of trespassers on border perimeters or moving objects in target areas, platforms with enhanced capabilities, like the one provided in this work, allow envisioning advance features like identity and location tracking, and foresee the use of WSNs to locate missing people, identifying criminals or detect relevant activities [11–14]. Personal care applications that use WSNs can also break with the current state-of-the-art, including actual telemedicine and a complete patient monitoring, above traditional monitoring of simple body’s parameters. For instance, in [15] a complete ECC monitoring scheme is shown. The non-intrusive study of people’s behavior, mainly of elder people suffering dementia, has also been reported in the state-of-the-art [16,17]. Regarding to environmental and industrial fields, high performance WSNs might be applied to support full manufacturing processes, including quality control relying on artificial vision techniques. On the other hand, gaming is an example of a novel application field, where 3D environmental virtualization with WSNs [18,19] allows the evolution from current networking games to future pervasive approaches.

The architectural changes of wireless sensor nodes are not only caused by the inclusion of new sensors, but also by the toughening of traditional node constraints such as maximum latency, number of nodes or bandwidth. Besides, the increase of security challenges has to be considered as well, as can be seen in [20,21], including the implementation of outstanding encryption algorithms, like ECC [22,23], which were originally considered unfeasible for constrained WSNs. For instance [24], deals with data-mining for WSNs while [25] addresses distribute multimedia source coding.

Nevertheless, introducing FPGAs in wireless sensor nodes to deal with high demanding scenarios is not a novel approach in the state-of-the-art. Thus, in [26] the authors introduced a Spartan 3E prototype board as a high performance coprocessor attached to an external ZigBee transceiver. The use case reported in the work was a hyper-chaos encryption engine. Similar approaches using off-the-shelf FPGA boards are offered in [27–30], this last one being oriented towards visual sensors. These previous approaches prove that including reconfigurable devices in WSNs offer certain benefits in terms of flexibility or performance. However, despite being valid for proof of concept demonstration, existing approaches are far from showing actual WSN node architectures, leaving out important issues such as power consumption, power management or the integration of sensor devices, among others. By contrast, the development of a complete FPGA based node is provided in [31], including a low performance Spartan 2E. The full node including the communication circuitry is integrated in a 25 mm × 25 mm board. The Cookies platform, which is the base of the processing layer shown in this work, follows the same approach [3] including a Spartan 3 or an Actel Igloo FPGA in different versions.

Regarding hardware reconfiguration, other solutions have been also proposed in the state-of-the-art. In [32], a reconfigurable node that includes a low-power flash-based FPGA is explained. The authors overcome the dynamic reconfiguration problems of the flash-based devices by having a virtual layer on top of the logic one, which enables different blocks of the system depending on the run-time needs. In this case, dynamic reconfiguration is emulated, since all the blocks already exist in the logic although they can be used or not. Furthermore, some approaches including Dynamic and Partial Reconfiguration using SRAM-based devices have been proposed. In [33], a platform called MicrelEye is proposed. This platform uses an FPSLIC configurable platform that includes a small (40k gates) FPGA and an ATMEL microcontroller. In spite of the small area of the device, this is an interesting approach, evidencing the flexibility offered by configurability, and its application to image sensing. In [34], a Virtex-4 FPGA is used to implement a dynamically reconfigurable sensor node. This work is focused on the internal design of the FPGA architecture, including a reconfigurable Kalman Filter to remove noisy samples during data acquisition. The results obtained show the suitability of this technique to deal with flexible requirements. However, details about the node architecture or dynamic and partial reconfiguration impact on power management or start-up time are not included in the work. Also the original Cookie platform has been featured with dynamic and partial reconfiguration, using an external processor, as shown in [35].

In this work, an integrated design of an SRAM FPGA based high performance node including not only a high performance FPGA, but also power management and monitoring circuitries, external memories and conditioning circuitry for external sensors is proposed. Besides, power management strategies and a dynamic and partial reconfiguration mechanism are also proposed, integrating efficiently dynamic reconfiguration features within the node. Actual measurements of the architectural performance are also shown, comparing them with WSNs traditional software based approaches for cryptographic applications.

## 3. Node Architecture: HiReCookie

As it was mentioned before, the adaptation of the Cookie WSN node to comply with the requirements imposed by high performance applications is faced in this work. In [3], the Cookies architecture is explained in detail. To summarize, some information about the node architecture is explained below. The Cookie node consists of four different layers:

- Sensing layer: it includes conditioning circuits for both digital and analog sensors. The output signal of these conditioning circuits goes through the vertical connectors to the processing layer. There are few different versions of this layer with different sensors: accelerometer, video camera, air temperature, humidity, strain gauge, carbon monoxide, carbon dioxide, oxygen, water temperature and pH.
- Power supply layer: it is the power source of the node. The node can be powered from an USB cable, lithium or AA batteries or directly from the mains if it is necessary.
- Communication layer: it includes the radio module to communicate data between nodes. It can be either a ZigBee or a Bluetooth module. In the case of the ZigBee module, different frequencies are available (2.4 GHz and 868 MHz).
- Processing layer: it is the *brain* of the platform. It is the layer in charge of processing all the information given by the sensors and the radio module. It includes an FPGA and a microcontroller that changes depending on each version. The first version of the processing layer of the Cookies includes a low-cost and low-performance Spartan 3 FPGA, together with an external microprocessor (ADuC841). In this previous version, the FPGA is in charge of interfacing with different sensors and carrying out simple preprocessing tasks. In turn, the external microprocessor executes the main node functions. Despite of the inclusion of an FPGA, the processing capabilities of the node were limited, and therefore, in this work its capabilities are extended. Finally, other version of the processing board including an ultra-low-power microprocessor (TI MSP430 family) and an ultra-low power FPGA (Actel Igloo) has been previously implemented, and is used as a reference in this work.

**Figure 2.**
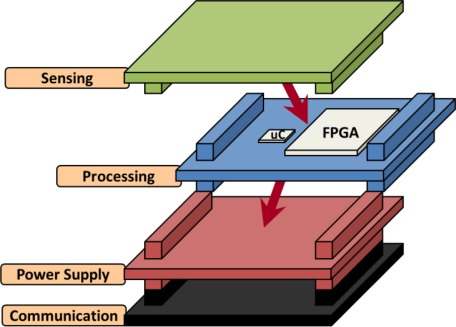
Cookies architecture.

The modularity of the platform can be seen in Figure 2. The vertical connectors have two main goals: acting as a join point between layers and connecting all the signals from one layer to the other. In this way, it is possible to change every layer separately if different sensors, communication modules, power supply sources, *etc*. are needed. This is one of the main features of this platform and can be very useful when adapting it to different requirements and scenarios. Thanks to this modularity, even though the processing layer is completely redesigned in this work, the others can be reused without further changes.

### 3.1. Processing Layer Architecture

The convenience of using hardware based systems to face those strict requirements was already discussed in previous sections. In this case, a Spartan 6 XC6SLX150-2 with FGG484 package is the one that better matches the requirements. This is a last generation FPGA with competitive power consumption and price. It has 147,447 logic cells in a package of 484 I/O ports divided into four banks, enough to place all the hardware modules and external peripheral controllers needed for the applications. Even though the speed grade chosen is (−2), the package is compatible with the lower power version with speed grade (−1) that can save up to 40% of the energy by only reducing 20% of the speed, according to the data given by the manufacturer (this will be implemented in future work).

#### 3.1.1. Flash Memory

A non-volatile memory has been included in the node, due to four main reasons:

- To store sensor data to be processed and sent through the network.
- To store program data to be used by the embedded processor.
- To work as the configuration bitstream source for the FPGA, after a switch off period.
- To store partial bitstreams corresponding to different hardware modules, which are configured in the device at run-time only when they are required.

According to the proposed power management strategies described in Section 4, all the components included in the node will be switched off during their sleep periods. One of the drawbacks of using an RAM-based FPGA is the fact that it does not keep its configuration when the power supply is cut off. Therefore, it is necessary to store an initial bitstream in a non-volatile device. In this case, a flash memory has been selected. In this way, using an automatic configuration mode (Master BPI Mode), the bitstream is automatically downloaded into the FPGA once both devices are powered on.

In order to work as the initial bitstream source for the FPGA, the memory must be connected to the dedicated configuration pins that are in banks 1 and 2. Nevertheless, the memory will also work as a storage device for other kind of information, so it needs to be accessible through another controller as well. Due to the restrictions imposed by the virtual architecture (the internal architecture within the FPGA), explained in the following section, the memory controller cannot be placed close to this I/O banks since it is within the reconfigurable area, so two different connections will be required for this memory.

The memory device selected is a Numonyx Strata flash Embedded Memory (P30) model JS28F128P30B85. This is a NOR flash memory recommended by Xilinx with 128 Mbits of capacity. It can be powered with 1.8 V which matches perfectly well with the rest of the power rails. The initial access time is 85 ns working at 52 MHz.

#### 3.1.2. RAM Memory

According to the methodology proposed in the present work, energy savings are increased by speeding up the processing. Due to this fact, a RAM memory has been also included in the node to allow fast information accesses by the embedded processor. Partial and dynamic reconfiguration speed can also have benefits by using an external RAM memory to store partial bitstreams, compared to the use of the external flash.

The memory device chosen is the Mobile Low-Power SDR SDRAM MT48H16M16LF from Micron. This memory is a high-speed 256 Mbits, CMOS dynamic random-access memory. The speed grade chosen is (−75) which corresponds to a 133 MHz with an access time of 5.4 ns. As well as in the case of the flash memory, it can be powered at 1.8 V.

#### 3.1.3. Analog to Digital Converter (ADC)

Analog to digital conversion is needed to process the signals from the sensors. Besides, the system has the ability of measure its own power consumption for each power island. The chosen ADC is the AD7928 from Analog devices. The AD7928 is an eight inputs device with a SPI interface to communicate with the FPGA. It is an 8-bit resolution converter with a throughput rate of 1 MSPS that can be powered at 3.3 V which matches the voltage ranges imposed by the sensors.

#### 3.1.4. Instrumentation Amplifier

In order to be able to measure power consumption of the system, every power island includes a shunt resistor (10 mΩ) where the drop voltage is measured and adapted by an instrumentation amplifier. These values will be converted by the ADC and then processed by the FPGA. The selected amplifier is the INA333 from Texas Instruments. This is a micro-power, zero-drift, rail-to-rail out instrumentation amplifier. The way in which the power islands are organized will be explained in the Power management section.

**Figure 3.**
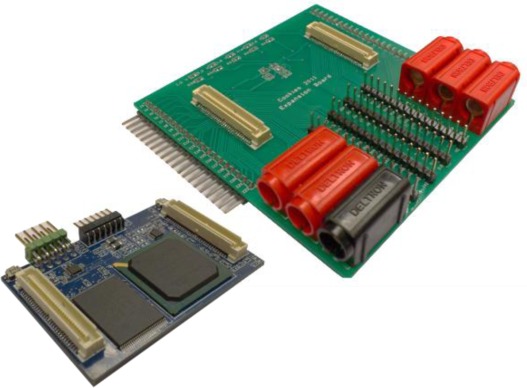
HiReCookie on the left side and expansion board for debugging on the right side.

The final PCB with all the components is shown in Figure 3. This is a 60 × 40 mm board composed of ten different layers and more than 150 decoupling capacitors, the majority of them 0201 package style. The board is a final prototype that matches with the physical requirements imposed by the sensor platform.

Testability is one of the most important requirements for the platform. The board includes two JTAG ports to test and program both the FPGA and the external microcontroller. Apart from that, an expansion board has been designed in order to have as much available connections as possible to be tested. This expansion board will be used for both, testing and powering, the platform in the lab.

Apart from the FPGA chosen, there is an external tiny microcontroller for energy management which will be detailed in the following section.

### 3.2. Virtual Architecture

**Figure 4.**
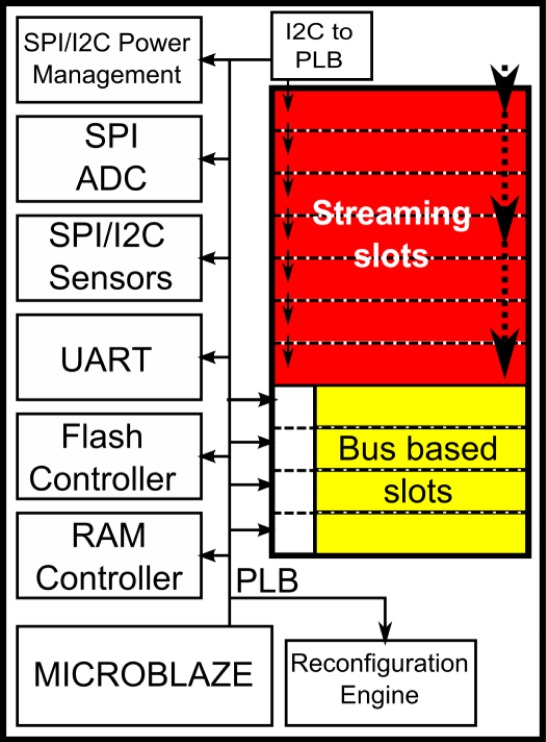
FPGA inner blocks. Virtual architecture.

After showing the general node architecture, further details about the FPGA internal virtual design are provided in this section. Virtual Architecture (VA) refers to the size and position of the reconfigurable areas, the communication among them, as well as between reconfigurable and static parts of the system. The term Virtual Architecture comes from the virtual memory concept, widely used in operating systems [36]. In dynamically reconfigurable system design, hardware virtualization allows the execution of complex applications on hardware platforms with insufficient resources. It also allows hardware module relocation between different virtual reconfigurable modules, and even between different reconfigurable systems with similar features. The HiReCookie VA has been defined as follows. The inner FPGA resources have been divided into two different regions: on the right side (bank 1), the reconfigurable area is used to allocate changeable blocks at run-time and on the left side (bank 3) the static area where the modules which remain unchanged during the system life-time are placed. This division can be seen in Figure 4. According to the results obtained with the Xilinx ISE tool, the static part occupies approximately 60% of the total area of the FPGA, leaving the other 40% for the reconfigurable blocks.

#### 3.2.1. Static Region

All the controllers required to interface with the external node components have been located in the static region. In addition, an embedded Microblaze processor working as the system controller has been implemented in this part. The processor will be in charge of executing software tasks within the node, since unlike the previous Cookie platform, a general purpose external microprocessor has not been included in the mote. The rest of the hardware controllers and the reconfigurable accelerators are connected to this processor using a Processor Local Bus (PLB), following a System on Chip (SoC) approach. Together with the Microblaze processor, the main blocks are detailed below:

- A reconfiguration module which makes use of the internal programming port (ICAP) to place reconfigurable blocks by writing into the associated areas of the reconfiguration memory of the FPGA. Further details of this block are included in Section 4.3.
- Memory controllers, for both RAM and flash. MPMC and EMC_MCH modules given by Xilinx.
- UART interface to communicate with the ZigBee communication module.
- SPI interface to communicate with both, the external microcontroller and the ADC.
- Several I2C and SPI peripheral modules for smart digital sensors interfacing.
- Chip-Scope peripheral for debugging issues.

The 60% area usage of the FPGA can be reduced by erasing the debug modules and some of the sensor controllers that may not be needed in all the applications.

#### 3.2.2. Reconfigurable Region

The remaining FPGA resources have been included in the reconfigurable section. In this area, different hardware accelerators used only during certain time periods or under specific application conditions can be allocated. Thus, dynamic reconfiguration allows the implementation of complex systems by means of resource sharing, as well as its functional adaptation after deployment.

**Figure 5.**
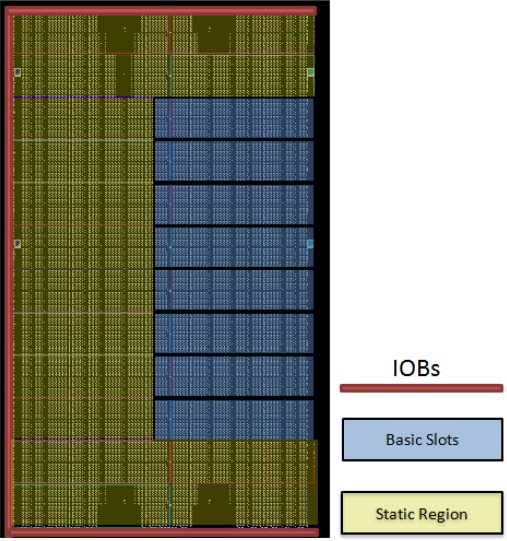
Resource utilization of the FPGA.

This area has been split up in several fixed regions called reconfigurable slots. As can be seen in Figure 5, some of these slots are connected using a streaming structure, while the others follow a bus-based approach. In both cases, those reconfigurable modules are connected with the static side using a bus-based approach. In order to dimension the reconfigurable slots, some blocks typically used in secure WSN applications have been tested. In Table 1, two encryption algorithms and their space utilization are shown. Those values are given by the Xilinx ISE tool once the blocks are located inside the FPGA. According to the defined size, every slot may contain up to 960 slices, 12 Block RAM modules and 8 DSP48E processing blocks.

According to these values, the area division is shown in Figure 5, where the reconfigurable slots, the static region and the IOBs used are shown. Notice that each slot spans a full clock region and usable IOBs have been located in the static region. In addition, no slots have been included both at the top and the bottom of the right side of the FPGA, since ICAP and BSCAN ports, which might be accessible from the static region, are located in these areas.

## 4. Power Management Strategies

The inclusion of high performance components implies an important handicap in terms of power consumption. Therefore, it is necessary to include very efficient power management strategies to comply with WSN requirements. Even though most of these components have their own power down modes, current consumption is still too high for a platform that needs to work unattended and powered by batteries. Power management strategies have a critical impact in three main situations:

1. During sleeping time: turning all the components unused off to achieve zero current consumption.
2. During FPGA Configuration time: making it as fast as possible by reducing the initial bitstream file.
3. During run time: doing the calculations as fast as possible and turning on only the components that are strictly needed.

The first of the proposed strategies is the use of power islands, which is the mechanism that allows switching on the devices included in the board, only when it is required by the application.

### 4.1. Power Islands

The architecture proposed in this work is divided into seven different power islands that can be switched on and off separately depending on the system needs. Therefore, all the components can be switched off during sleeping time so that power consumption is sharply decreased. In order to control these islands, it has been necessary to include another component acting as a sentry to wake the system up when either a measurement or a calculation need to be done. The component chosen to carry out this duty is a tiny AVR microcontroller (ATtiny 2,313V). This is a 2 kb flash controller with very low price and size, and less than 0.1 μA of current consumption during power down mode. This tiny processor will control the enables of seven different power switches to master the island supplies. This component will not be in charge of taking any autonomous decisions. The FPGA informs the ATtiny about what to do before switching the islands off. Every island includes a shunt resistor for its instant power consumption monitoring. The power islands implemented are listed below:

- Island 1: FPGA core. Powered at 1.2 V.
- Island 2: Sensor and communication boards. Powered at 3.3 V.
- Island 3: ADC and power consumption circuitry. Powered at 3.3 V.
- Island 4: RAM memory and bank 3 of the FPGA. Powered at 1.8 V.
- Island 5: Flash memory and Banks 1, 2 of the FPGA. Powered at 1.8 V.
- Island 6: External clock and Bank 0 of the FPGA. Powered at 3.3 V.
- Island 7: Auxiliary logic of the FPGA. Powered at 2.5 V.

As it can be seen, four different power supply voltages are used: 1.2 V, 1.8 V, 2.5 V and 3.3 V. The FPGA core is the only one powered at 1.2 V and, therefore, it needs to be in an independent island. The 1.8 V supply is used to power banks 1, 2 and 3, the external memories and the external microcontroller. The external microcontroller is not included in the islands division since it needs to be to be always powered. During the initial configuration, the bitstream is downloaded from the flash memory through the dedicated configuration pins which are within banks 1 and 2. Due to this reason both the flash memory and banks 1 and 2 belongs to the same island. The RAM memory and bank 3 are placed together in a different island, because the RAM memory controller, which is a hard IP of the FPGA, is located on the left side. In the case of the 2.5 V supply, it needs to be turned on at every time the core is powered. This auxiliary logic is not included in the island of the core because the power supply value is different and because it is necessary to have separated power consumption measurements for both rails. Regarding the 3.3 V rail, independent islands have been included to allow switching the sensor and communication layers separately from the power consumption circuitry.

### 4.2. Initial Bitstream Compression

The bitstream file is stored in the flash memory device, to carry out the initial reconfiguration after a switch off period. The size of that bitstream is very significant in terms of power consumption, since it is directly connected with the configuration time.

In Table 2, sizes of two different bitstreams are shown. In the case of the compressed one, the reduction is done directly by the Xilinx tool with 60% of compression. Even though this is an important reduction, the compression can be still optimized. In this work, a methodology to improve the compression of the bitstream file has been also included, using similar strategies to those reported in [8].

**Figure 6.**
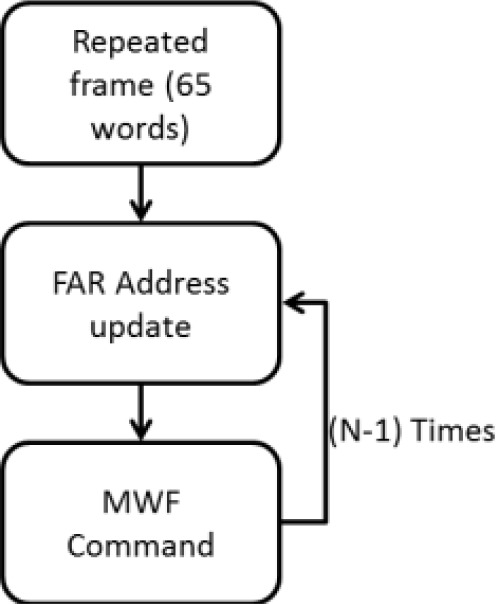
Compression algorithm.

The followed approach is based on the fact that the FPGA is empty by default, that is, the bitstream is full of zeros. In this way, it is possible to erase them so that the size of the bitstream would be reduced without losing any information. The start point for this optimization can be either a total or a compressed bitstream. A basic configuration unit of the FPGA is called frame. Every frame consists of 65 words, so if the same frame is to be written a fixed number of times, it is quite consuming in terms of space. However, in the case of the compressed one, when a frame is repeated a certain number of times, the frame is stored in a register called Multiple Write Frame Register (MWFR) followed by a command to specify the frame address (FAR address). The procedure is shown in Figure 6.

Considering the structure of the compressed bitstream, the approach to reduce configuration time is to identify the empty frames and to erase the MWF commands. This strategy deletes all the redundant zeros regarding the CLBs information. The MWF command is not used for the BRAMs configuration, so it will be necessary to check frame by frame to erase the empty ones. These times have been measured using an oscilloscope.

### 4.3. Dynamic and Partial Reconfiguration Engine

Apart from its possibilities in terms of flexibility, dynamic and partial reconfiguration (DPR) can be used to reach an energy efficient system. Efficiency is obtained by means of DPR thanks to resource sharing, but also considering that only those hardware modules required at each moment have to be configured in the device. Furthermore, more optimal hardware can be designed, if it is specifically done focusing on each situation, instead of addressing general purposes. In previous works [37], the authors have already provided a reconfiguration engine for Spartan 6, which exploits reconfiguration capabilities using the Internal Configuration Access Port (ICAP) of the FPGA. The main feature of the block is its relocation capability, which means, the possibility of changing at run time the position of the reconfigurable blocks within the device. Thus, it is possible to allocate each reconfiguration engine in any empty slot in the reconfigurable region.

### 4.4. Power Management Scenarios

The external processor will be in sleep mode (using its power save modes) until an external interruption or a timer wakes it up. The FPGA is linked to the ATtiny through a SPI connection. In this way, the embedded Microblaze can send the needed commands to inform the external microcontroller how it must be awakened before entering into sleep mode. A complete set of functions has been designed to define this communication. Therefore, the management of the islands can be selected only by changing the code of the Microblaze processor which is included in the bitstream file. The foreseen wake-up options are: an interrupt from the communication module, a sensor value bigger than certain threshold and timers. This way, different mechanisms have been implemented, as listed below:

- Scenario 1: before entering into sleep mode, the embedded processor sends a command through the SPI connection to order the ATtiny to wake the system up in case an interrupt from the radio is received. Then, the ATtiny switches all the islands off, including the FPGA, and enters in power down mode waiting for the external interruption to occur. During this period, only the external microcontroller, the communication module and the power supply board are powered on, being the power consumption 9 mA.
- Scenario 2: before entering into sleep mode, the FPGA sends a command to the ATtiny to request switching on the communication module periodically in order to check if an interrupt from the radio arrives. Then, the ATtiny switches all the islands off and enters in power down mode waiting for the timer to occur. Until an external interrupt happens, the microcontroller is in power down mode and the communication module is also powered off, only the ATtiny and the power supply board are powered on, so the power consumption is in the order 10 μA.
- Scenario 3: the communication module also has power down modes. In this way, it is possible to have the same case as in scenario 2 but instead of switching the module on and off, the ATtiny can change the mode periodically to check if the radio interrupt occurs. In this case, the power supply board, the ATtiny and the communication module are powered on, but the communication module is in power down mode as well as the ATtiny, so the power consumption is in the order of 11 μA.
- Scenario 4: before entering into sleep mode, the Microblaze sends a command to the ATtiny to wake the system up in case an interrupt request from the analog comparator occurs. Then, the ATtiny switches all the islands off and enters in a sleep mode waiting for the external interrupt to occur. During this period, if the value in one of the analog sensors is bigger than the programmed threshold, the ATtiny wakes up so it can switch on any island needed. In this case, only the external microcontroller, the sensors layer and the power supply board are powered on, so the power consumption depends on the sensors used.
- Scenario 5: before entering to sleep mode, the Microblaze sends a command to the ATtiny to request waking the system up periodically. Then, the ATtiny switches all the islands off and enters into a sleep mode until the timer interrupts. During this period, only the external microcontroller is in power down mode, and the power supply board is powered on, so the power consumption is in the order of 10 μA.

Once the system is recovered from sleep mode, the ATtiny can switch on only the islands that are strictly needed depending on the application, so the consumption can be also optimized during active time. Besides, when the FPGA is powered on, the ATtiny sends a register including the information of what is already powered on and why the system was powered on so that the FPGA can know all the information about the status of the rest of the components.

## 5. Platform Test and Validation

**Figure 7.**
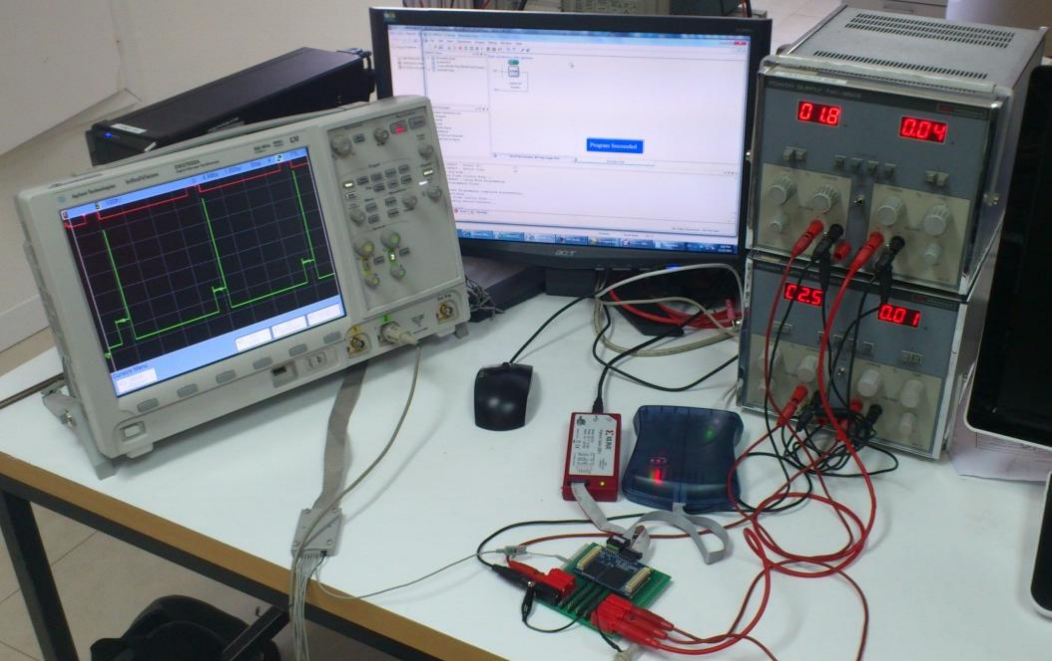
Test setup.

In this section, different tests and results are to be detailed. The purpose is to provide, by means of well-known application examples, both the computation power of the proposed mote, as well as its energy efficiency when facing very intensive computational tasks. The setup used for these tests can be seen in Figure 7. In order to obtain the consumption profile, an oscilloscope has been used to monitor the current consumption of the FPGA core using the output signal of the instrumentation amplifier of the first power island. The curves shown in this chapter represent only the power consumption of the FPGA core, so both the consumption of the auxiliary logic and the IOBs must be added. The consumption of these power islands is also monitored through the SPI block connected to the ADC. Therefore, the values to be added during run-time are 30 mA during configuration and 40 mA during computing time. During sleeping-time, all the islands are powered off, so the power consumption is 0 A in all the cases. As it can be seen on the figures, the value of the current consumption when the system is not powered has an offset. It appears because all the power measurements are done using the instrumentation amplifier. The offset of this amplifier is 21 mV when the current consumed by the island is 0 A.

The selected modules used to run on the FPGA are the SHA1 and MD5 encryption algorithms. Those algorithms are extendedly used in wireless environments to check message integrity and authentication. These algorithms are used as examples for the platform validation because they are algorithms used in real applications for secure WSNs. This way, real needs can be tested to demonstrate the feasibility of the theoretical proposals.

Both SHA1 and MD5 are implemented as independent hardware blocks and as software code running on the embedded processor. In both cases, the number of data blocks to be encrypted is configurable. This way, it is possible to evaluate the minimum number of data blocks from which hardware starts being worthy compared with software. As it will be seen below, the time needed to configure the FPGA with the initial bitstream is crucial to minimize power consumption, since this time is huge compared with the one needed for calculations.

Apart from different data blocks and bitstreams, these tests will also combine different wake-up policies such as the ones seen on the power management strategies section.

These validation tests show the high flexibility of the platform. The possibility of running different algorithms in hardware and software at the same time depending on each application scenario gives the chance of optimizing task scheduling and power efficiency. Besides, the debugging capabilities compared with previous versions of the Cookie node are drastically increased. The use of the JTAG port, both for hardware and software debugging, the use of the ChipScope module, or even the UART interface, have been essential for the correct development of the applications and lab tests.

All the different combinations are to be compared, while the same algorithms will be also tested in a low power version of the Cookie node that includes an MSP430 microcontroller.

The name of the different tests is coded as follows (1–5):

1. **SHA1** or **MD5** encryption algorithm.
2. **HSW** if the encryption algorithm is running on the platform Microblaze. **HW** if the encryption algorithm is running as a hardware block on the FPGA. **SW** if it is running on the MSP430 processor, in the low power consumption version of the Cookies.
3. Number of data blocks to be encrypted. Each block corresponds to 16 32-bit row data words.
4. **COMP** if a compressed bitstream is being used or **TOT** in the case of the non-compressed one.
5. Depending on the wake-up policy used: **ZB** for the radio, **TIMER(x)** if it is using the timer every x seconds.

All the tests shown have been carried out using the final prototype facing real requirements in real cases. These results validate the theory shown in the introduction section, Figure 1.

### 5.1. SHA1 Module Testing

In this application scenario, the node is in sleep mode and awakening only for the encryption of a variable number of data blocks. All the different cases are shown as follows. Notice that power consumption in every figure is given in mV. This value must be divided by the gain of the instrumentation amplifier which is 3.22.

### TEST 1: (SHA1, HSW, 128, TOT, TIMER (5))

**Figure 8.**
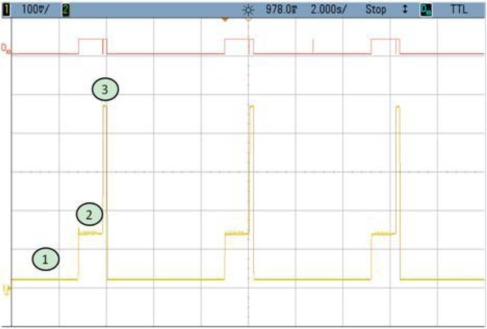
Test 1 SHA1.

The power consumption profile of the FPGA core is shown in Figure 8. On this oscilloscope image, the system is awakened every 5 s to encrypt 128 data blocks using the SHA1 algorithm. The time interval labeled 1 show the sleeping time of the node. The second period corresponds to power consumption during configuration, while the power consumption peak represents the actual FPGA activity. Numerical results are detailed in Table 3. Even though the current peak is quite high, the power supply board uses an integrated DC to DC converter from Texas Instruments (TPS650243) which is capable of giving up to 1.6 A. Nevertheless, for these tests, as it was mentioned before, an expansion board and external power supplies have been used. The wave form labeled as D10 shows the triggers to measure the computing and configuration times.

This example shows how critical the configuration time can become. In this case this time is more than twice the value during computing time. Therefore, the main goal of the following tests is to improve this configuration by reducing the size of the initial bitstream as much as possible.

### TEST 2: (SHA1, HSW, 128, COMP, TIMER (5))

**Figure 9.**
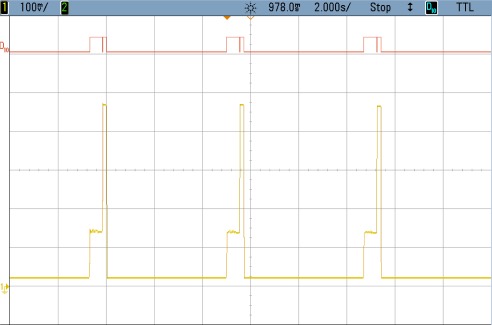
Test 2 SHA1.

The power consumption profile of the FPGA core for this test is shown in Figure 9. This case includes a compressed bitstream instead of a complete one. Numerical results are detailed in Table 4.

In this case the power consumption during configuration time is halved due to the bitstream compression while the other areas remain unchanged. This is translated into important savings compared with the previous case (TEST 1).

### TEST 3: (SHA1, HSW, 2000, COMP, TIMER (5))

**Figure 10.**
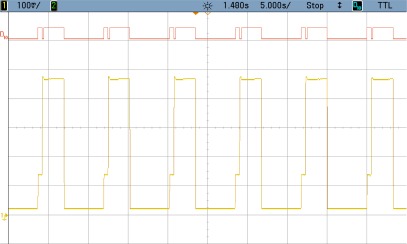
Test 3 SHA1.

By changing the number of data blocks to be encrypted each time the FPGA is awakening, the convenience of using hardware against software is studied. Therefore, Figure 10 shows the power consumption profile of the FPGA core where 2,000 blocks are processed instead of 128. Numeric results are shown in Table 5.

The enlargement of the consumption peak in the previous image shows how the encryption of 2,000 data blocks takes an extremely long time of 2.73 s, when SHA1 software code developed for the Microblaze is used. This implies a non-affordable power consumption of 140 mA during a long period of time. This computing time is sharply reduced using the hardware block, as it will be shown in the next test case.

### TEST 4: (SHA1, HW, 2000, COMP, TIMER (2))

**Figure 11.**
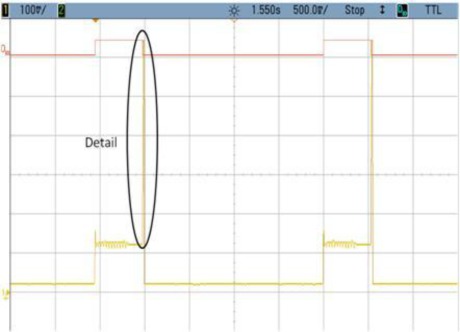
Test 4 SHA1.

**Figure 12.**
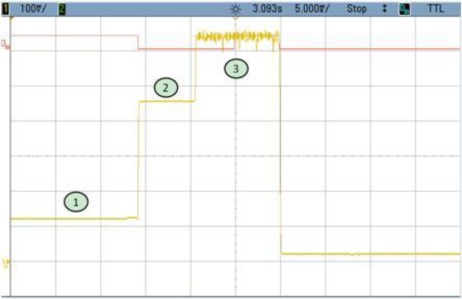
Test 4 SHA1 Detail.

The consumption profile is shown in Figure 11. In this example unprocessed data buffers are created by the Microblaze. That is the reason why zone n° 2 in Figure 12 appears. This time could be reduced by loading directly the hardware module and therefore decreasing the power consumption at run-time. Even having this additional time, the results are clearly improved compared with TEST 3, since power consumption per cycle is 15 times less in the case of Test 4.

**Figure 13.**
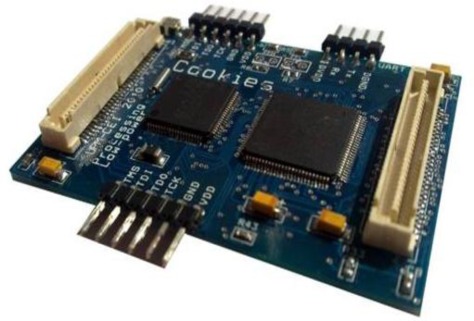
MSP430 cookie processing layer.

The comparison between hardware and software when software is running in this platform is now clear. Nevertheless, software tests are still running inside the FPGA, so configuration time is also required. In addition, using an embedded Microblaze inside a high performance FPGA is a completely inefficient choice when only software is required. Thus, for the sake of correctness, SHA1 and MD5 have been also ported to a Cookie with a MSP430 microcontroller shown in Figure 13. This microcontroller is widely used in WSN like the TelosB platform [2]. Numeric results can be seen in Table 6.

### TEST 5: (SHA1, SW, 2000, -, -)

**Figure 14.**
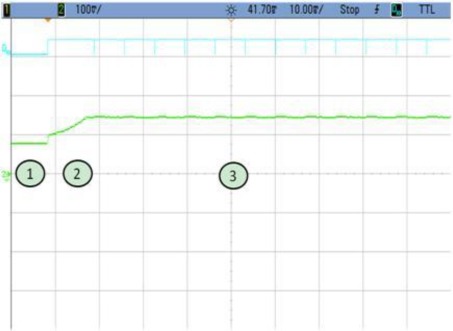
Test 5 SHA1.

In Figure 14, the microcontroller consumption profile is shown. The time necessary for the MSP430 to encrypt a data block is 7 ms. Time and power consumption to process 2000 and 10000 data blocks are shown in Table 7.

These tests, using low power Cookies, have been carried out with the MSP430 running at 4 MHz and activating low power mode during sleep time (period 1 in Figure 14).

### TEST 6: (SHA1, HW, 10000, COMP, ZB)

**Figure 15.**
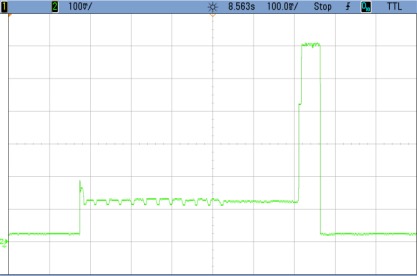
Test 6 SHA1.

**Figure 16.**
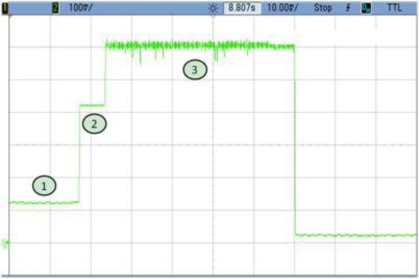
Test 6 SHA1 Detail.

In the case of the encryption of 10,000 blocks using hardware implementation of the SHA1, all the results are shown in Figures 15 and 16.

Tables 7 and 8 show a comparison between both encryption algorithms. Even though SHA1 and MD5 are not very complex algorithms, for enough block sizes, the platform proposed in this work beats state-of-the-art WSN microcontrollers in terms of power efficiency.

### 5.2. MD5 Module Testing

**Figure 17.**
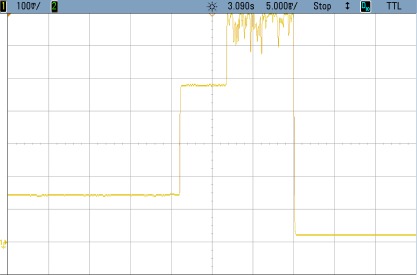
Detail (MD5, HW, 2000, COMP, -).

**Figure 18.**
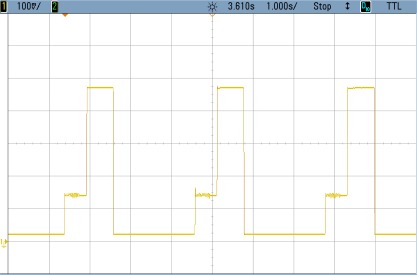
Detail (MD5, SW, 2000, COMP, -).

Apart from the SHA1 algorithm, the same tests have been done using the MD5. Two examples are shown in Figure 17 and Figure 18 to illustrate the comparison. The measurements in Table 9 show the execution time for both MD5 and SHA1 encryption engines in different cases. This information is also valid for the previous tests since the rest of the periods are approximately the same. When the application has to be adapted, like in this case, changing the encryption algorithm, thanks to the DPR feature of the node, not only software can be changed after deployment. Also hardware modules, which have been proved to be more efficient, can be easily loaded depending on the system needs at run time.

## 6. Conclusions and Future Work

In this work a high performance node architecture for demanding WSN applications based on a commercial SRAM-based FPGA is proposed. FPGA reconfiguration features increase node flexibility, while its hardware nature reduces execution times. Considering the periodic nature of typical WSNs’ tasks, together with power management strategies also proposed in this work, high power efficiency is achieved. Experiments have been carried out to validate the node behavior, as well as to quantify the benefits of including hardware components, compared with state-of-the-art low-performance software based solutions.

In the future work will be carried out in order to improve power management strategies, considering triggers coming from the radio interface, among others. Different sensor layers will be also attached to the platform, including a low-power CMOS camera to develop more advanced applications. More specifically, the objective is to develop a secure smart surveillance system, using a heterogeneous wireless sensor network topology. Each node will include video compression algorithms, both public and private key cryptography engines, as well as different scalar sensors.

## Figures and Tables

**Table 1.** Resource utilization of the different Encryption Algorithms.

**Resources**	**SHA1**	**MD5**
DSP48Es	3 out of 180 (1%)	3 out of 180 (1%)
Block RAMs	4 out of 268 (1%)	8 out of 268 (2%)
Number of occupied slices	753 out of 23,038 (3%)	401 out of 23,038 (1%)

**Table 2.** Initial bitstream comparison.

**INITIAL CONFIGURATION**	**Size (Kb)**	**Time**
Total bitstream	4,122	1.002 s
Compressed bitstream	1,416	353 ms

**Table 3.** Test 1.

**TEST 1**	**Current (mA)**	**Time (s)**	**Energy mAh**
1.Off-time	0	5	0
2. Configuration time	50	1	0.014
3.Computing time	140	0.16	0.006
Total per cycle	-	-	0.020

**Table 4.** Test 2.

**TEST 2**	**Current (mA)**	**Time (s)**	**Energy mAh**
1.Off-time	0	5	0
2. Configuration time	50	0.52	0.007
3.Computing time	140	0.16	0.006
Total per cycle	-	-	0.013

**Table 5.** Test 3.

**TEST 2**	**Current (mA)**	**Time (s)**	**Energy mAh**
1.Off-time	0	5	0
2. Configuration time	50	0.52	0.007
3.Computing time	140	2.73	0.106
Total per cycle	-	-	0.113

**Table 6.** Resource utilization of the different Encryption Algorithms.

**TEST 2**	**Current (mA)**	**Time (s)**	**Energy mAh**
1.Off-time	0	5	0
2. Configuration time	50	0.52	0.007
3.Computing time	134.5	0.0057	0.0002
Total per cycle	192	0.0093	0.0005
1.Off-time	-	-	0.0077

**Table 7.** Test 5.

**TEST 5**	**Current (mA)**	**Time (s)**	**Energy mAh**
1.Sleep mode	1.6	2	0,001
2.Wake-up time	2.4	0.01	6.7 × 10^−6^
3.Active mode (2000)	3	13.92	0.012
3.Active mode (10000)	3	69.6	0.058
Total value (2000)	-	-	0.013
Total value (10000)	-	-	0.059

**Table 8.** Test 6.

**TEST 2**	**Current (mA)**	**Time (s)**	**Energy mAh**
Off-Time	0	3.5	0
1. Config. Time	30.6	0.537	0.005
2. Activation Time	123	0.0064	2 × 10^−4^
3. computing Time	178	0.0465	0.002
Total per cycle	-	-	0.007

**Table 9.** MD5 *versus* SHA1.

**Comparison**	**Time (ms)**
SHA1 HSW	2.7
MD5 HSW	1.3
SHA1 HW	9.3
MD5 HW	8
